# The conjugation of nonsteroidal anti-inflammatory drugs (NSAID) to small peptides for generating multifunctional supramolecular nanofibers/hydrogels

**DOI:** 10.3762/bjoc.9.104

**Published:** 2013-05-10

**Authors:** Jiayang Li, Yi Kuang, Junfeng Shi, Yuan Gao, Jie Zhou, Bing Xu

**Affiliations:** 1 Department of Chemistry, Brandeis University, 415 South Street, Waltham, MA 02454, USA

**Keywords:** self-delivery, hydrogel, multifunctional, nanofibers, NSAID, self-assembly, supramolecular, topical use

## Abstract

Here we report supramolecular hydrogelators made of nonsteroidal anti-inflammatory drugs (NSAID) and small peptides. The covalent linkage of Phe–Phe and NSAIDs results in conjugates that self-assemble in water to form molecular nanofibers as the matrices of hydrogels. When the NSAID is naproxen (**1**), the resultant hydrogelator **1a** forms a hydrogel at a critical concentration (cgc) of 0.2 wt % at pH 7.0. Hydrogelator **1a**, also acting as a general motif, enables enzymatic hydrogelation in which the precursor turns into a hydrogelator upon hydrolysis catalyzed by a phosphatase at physiological conditions. The conjugates of Phe–Phe with other NSAIDs, such as (*R*)-flurbiprofen (**2**), racemic flurbiprofen (**3**), and racemic ibuprofen (**4**), are able to form molecular hydrogels, except in the case of aspirin (**5**). After the conjugation with the small peptides, NSAIDs exhibit improved selectivity to their targets. In addition, the peptides made of D-amino acids help preserve the activities of NSAIDs. Besides demonstrating that common NSAIDs are excellent candidates to promote aromatic–aromatic interaction in water to form hydrogels, this work contributes to the development of functional molecules that have dual or multiple roles and ultimately may lead to new molecular hydrogels of therapeutic agents for topical use.

## Introduction

This article reports the design, synthesis, and characterization of hydrogelators made of non-steroidal anti-inflammatory drugs (NSAID) and small peptides for the development of multifunctional supramolecular hydrogels. Sharing similar features with the extracellular matrices of tissues (e.g., consisting of three-dimensional networks and a significant amount of water), hydrogels have become an attractive choice for developing biomaterials for a variety of applications. For example, hydrogels have frequently served as scaffolds for tissue engineering and as carriers for drug delivery [1–4]. In the applications of drug delivery, it is common to use hydrogels made of biodegradable polymers to encapsulate therapeutic agents for controlled release of drugs by adjusting the pore sizes and functionality of the polymer networks [5–6]. Despite the tremendous success of this approach, several inherent shortcomings still limit the application of polymeric hydrogels. For example, the degradation of the polymers usually generates acidic monomers, which, sometimes, cause undesired inflammatory responses [7]. In most of the cases, the polymers themselves are passive, which unavoidably results in the limited loading of drug molecules [8–9]. Though possible, it is relatively difficult to functionalize the polymers with drug molecules. Even though it has been attempted, the release of the drugs from the polymer backbone remains a nontrivial issue. Despite being constantly improved, these limitations call for the exploration and development of new functional biomaterials and therapeutics.

The recent development of supramolecular nanofibers and hydrogels [10–25] presents an exciting opportunity for developing new types of biomaterials [26–29]. While the early works have centered on the use of nanofibers of oligopeptides to form hydrogels as a passive scaffold for tissue engineering and drug delivery [30], the seminal work by Stupp et al., in which functional hydrogels based on supramolecular nanofibers regulate the differentiation of neural progenitor cells [31], has demonstrated undoubtedly the power and promises of the rational incorporation of functional motifs into the building blocks of the supramolecular nanofibers and hydrogels. Besides being used for three-dimensional cell cultures [32], supramolecular hydrogels also find applications in typing [33] and against bacteria [34–38], and in enzyme assays [23,39], catalysis [40], and inhibiting cancer cells [41–44]. These successful examples further underscore the potentials of functional supramolecular hydrogels as new and useful biomaterials.

Since the formation of supramolecular hydrogels relies on the small molecules (i.e., the hydrogelators) that self-assemble in water through noncovalent interactions [45–46], these hydrogels are inherently easily biodegradable. To meet the prerequisites for self-delivery systems, besides biodegradability, the hydrogelators should preserve the pharmacological efficacy of the therapeutic building blocks, any side effects should be minimized and the biocompatibility maximized. Therefore, a convenient starting point for developing the molecular hydrogels of therapeutic agents is to construct hydrogelators only consisting of the therapeutic agent and biocompatible motifs [47–48]. Among a large pool of therapeutic agents, we choose to develop the molecular hydrogels of NSAIDs because topical use of the hydrogels of NSAIDs is an attractive therapeutic option for treating acute pain [49].

**Scheme 1:**
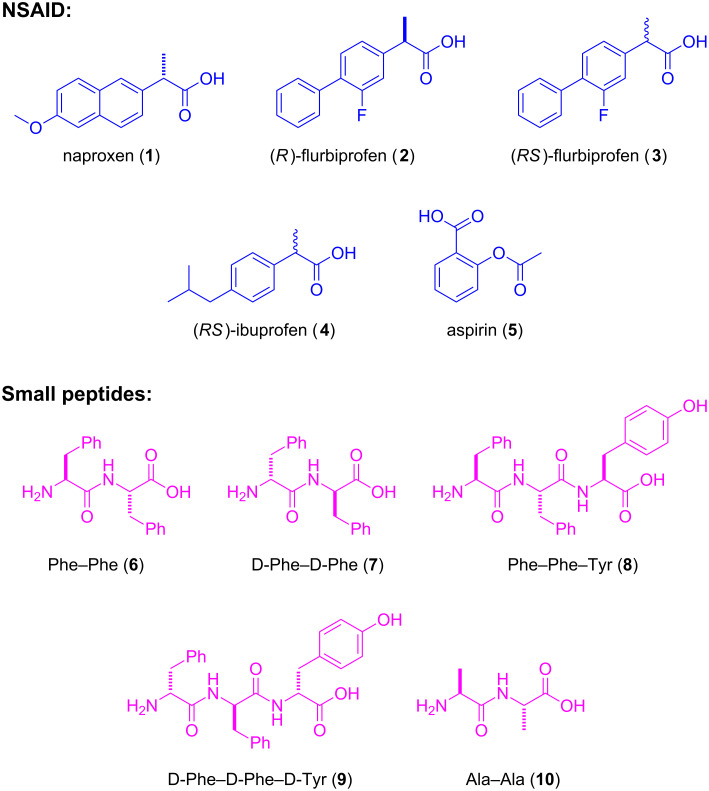
The structures of the NSAIDs and peptides explored as the building blocks of hydrogelators in this work.

Because of their analgesic, antipyretic, and anti-inflammatory effects (when being used in high dosage), NSAIDs are widely used for the treatment of acute or chronic pain or inflammations. Despite the widespread systemic use of NSAIDs, the adverse gastrointestinal and renal effects and cardiovascular risks associated with NSAIDs have led to the development of topical NSAIDs formulations, such as patch, gel, and solution [50–52]. Encouraged by the successful FDA approval of diclofenac gel [53] and several reports of small molecule hydrogels formed by NSAID derivatives [48,54–56] we intend to explore supramolecular hydrogels of other NSAIDs. Specifically, we use naproxen (denoted as Npx in this report), an over-the-counter NSAID, to generate a new hydrogelator **1a** that only consists of naproxen and diphenylalanine (FF). We use diphenylalanine because it is able to self-assemble to form nanotubes [57]. We find that compound **1a**, forming a hydrogel at the critical gelation concentration (cgc) of 0.2 wt % at pH 7.0, also serves as a general motif to enable enzymatic hydrogelation that converts **1c** to **1d** by a phosphatase and results in a hydrogel of **1d** under physiological conditions. In addition to naproxen, we evaluate the abilities of other NSAIDs (Scheme 1) as building blocks of hydrogelators and find that the conjugates of FF and (*R*)-flurbiprofen (**2**), racemic flurbiprofen (**3**) or racemic ibuprofen (**4**) form supramolecular hydrogels. Moreover, our previous examination of the efficacy of the hydrogelators derived from naproxen shows that the conjugation of small peptides may improve the selectivity of naproxen for inhibiting COX-2 and thus reduces their adverse effect [58]. Although the inhibition efficacies of the hydrogelators for both enzymes COX-1 and COX-2 are lower than naproxen, the use of D-peptide for conjugation helps preserve the activities of naproxen. These results provide useful insights for the optimization of these hydrogelators. Besides serving as a potential alternative approach to polymeric hydrogels [59–60], this work contributes to the development of bioactive molecules that have dual or multiple roles, for example, as therapeutic agents and delivery carriers [61–62].

## Results and Discussion

### Synthesis

**Scheme 2:**
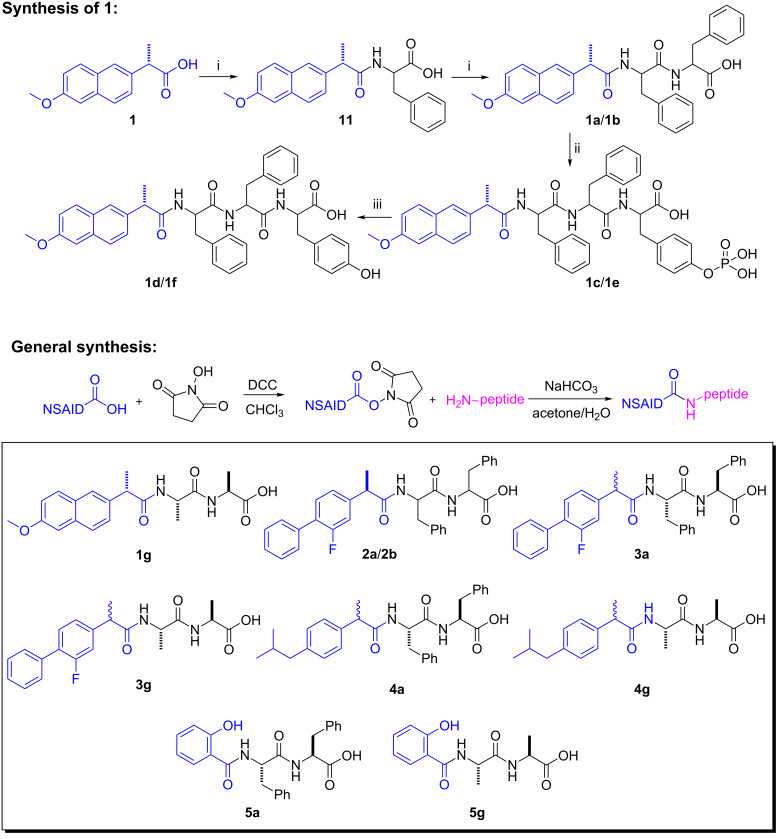
The synthetic route of the naproxen-containing hydrogelators and the corresponding precursors: (i) NHS, DCC, CHCl_3_, 2 h; Phe, pH 8.5, 12 h. (ii) NHS, DCC, CHCl_3_, 12 h; Tyr(p), pH 8.5, 24 h. (iii) alkaline phosphatase, pH 7.6.

Scheme 2 shows the typical synthetic route of the conjugates of NSAID and small peptides exemplified in the case of naproxen. In this process, naproxen (**1**) first reacts with *N*-hydroxysuccinimide (NHS) and *N*,*N*'-dicyclohexylcarbodiimide (DCC) in chloroform to afford the NHS ester of **1**, which reacts with a phenylalanine in the mixed solvent of acetone and water to produce **11**. The same coupling procedure allows the covalent attachment of second phenylalanine on the C-terminal of **11** to give **1a**. Being activated by NHS, **1a** reacts with phosphotyrosine (pY) to yield **1c** after purification by HPLC. Using the same procedure, we connect naproxen to D-peptides to make Npxff (**1b**), Npxffy(p) (**1e**), and Npxffy (**1f**), because D-amino acid based hydrogelators are able to resist proteases [63].

Scheme 2 shows the general synthetic route of other NSAID containing hydrogelators and lists all the NSAID-based conjugates studied in this work. Generally, after reacting with NHS and DCC in chloroform, the NSAID becomes a NHS ester, which reacts with the small peptides (e.g., Phe–Phe, Phe–Phe–Tyr, or Ala–Ala) in the mixed solvent of acetone and water. We have produced these NSAID-containing hydrogelators with yields from 40% to 60% after purification by HPLC or column chromatography. For example, flurbiprofen (*R*-enantiomer **2** or racemic **3**) conjugates with L- or D-diphenylalanine to afford compounds (*R*)-Fbp-FF (**2a**), (*R*)-Fbp-ff (**2b**), and (*RS*)-Fbp-FF (**3a**) (Fbp = flurbiprofen). The attachment of racemic ibuprofen to L-diphenylalanine yields compound (*RS*)-Ibp-FF (**4a**) (Ibp = ibuprofen). Since the direct use of acetylsalicylic acid (aspirin) to connect with dipeptides results in a compound hydrolyzing easily at the ester bond of aspirin, we use salicylic acid (Sal) instead to conjugate with diphenylalanine and dialanine (AA) to make compounds **5a** and **5g**, respectively.

### Self-assembly and hydrogelation

**Figure 1:**
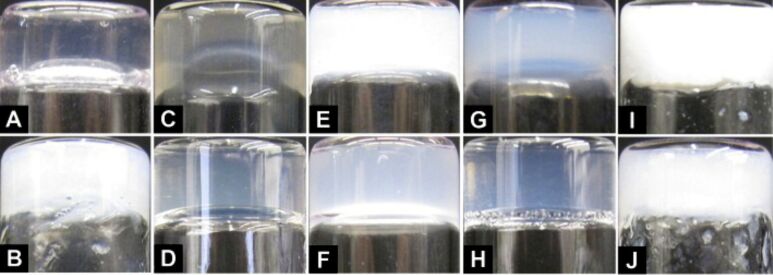
Optical images of the hydrogels formed by (A) **1a** (0.8 wt %, pH 7.0); (B) **1b** (0.8 wt %, pH 4.0); (C) **1d** (1.5 wt %, pH 7.6), obtained by treating **1c** with alkaline phosphatase (5.0 U/mL); (D) **1f** (0.8 wt %, pH 7.6), obtained by treating **1e** with alkaline phosphatase (5.0 U/mL); (E) **2a** (0.8 wt %, pH 7.2); (F) **2b** (0.8 wt %, pH 7.2); (G) **3a** (0.8 wt %, pH 7.2); (H) **4a** (0.8 wt %, pH 7.2); (I) **1g** (0.8 wt %, pH 4.0); (J) **3g** (0.8 wt %, pH 1.0).

After the synthesis of the NSAID/small-peptid conjugates, we examined the capability of the conjugates to self-assemble in water to form supramolecular nanofibers and hydrogels (Table 1). Typically, the addition of 8.0 mg of Npx-FF (**1a**) into 1.0 mL of water under basic conditions (pH 9.0) affords a solution, which turns into a suspension at pH 7.0. Upon heating to 60 °C, the suspension turns to a clear solution, and then becomes a transparent hydrogel after being cooled to room temperature (Figure 1A). The treatment with a small amount of base prior to the adjustment of pH appeared to be a necessary step for making the hydrogel. Being thermally reversible and relatively sensitive to the temperature, the hydrogel of **1a** exhibits excellent recovery properties (Supporting Information File 1, Figure S4) [64].

**Table 1:** The properties of the NSAID-containing hydrogels.

	concentration (wt %)	pH	method of preparation	components	gel properties

**1a**	0.8	7.0	heat–cool	naproxen, L-Phe–L-Phe	transparent gel, thermal reversible, mechanical recovery
**1b**	0.8	4.0	acidic pH	naproxen, D-Phe–D-Phe	opaque hydrogel, precipitate at high T,
**1d**	1.5	7.6	enzyme	naproxen, L-Phe–L-Phe–L-Tyr	transparent gel, mechanical recovery
**1f**	0.8	7.6	enzyme	naproxen, D-Phe–D-Phe–D-Tyr	transparent gel
**2a**	0.8	7.2	heat–cool	(*R*)-flurbiprofen, L-Phe–L-Phe	slightly opaque hydrogel, thermal reversible, weak mechanical recovery
**2b**	0.8	7.2	heat–cool	(*R*)-flurbiprofen, D-Phe–D-Phe	semitransparent hydrogel, thermal reversible, weak mechanical recovery
**3a**	0.8	7.2	heat–cool	(*RS*)-flurbiprofen, L-Phe–L-Phe	semitransparent hydrogel, thermal reversible, weak mechanical recovery
**4a**	0.8	7.2	heat–cool	(*RS*)-ibuprofen, L-Phe–L-Phe	semitransparent hydrogel, thermal reversible, weak mechanical recovery
**1g**	0.8	4.0	acidic pH	naproxen, L-Ala–L-Ala	opaque hydrogel, poor stability at rt
**3g**	0.8	1.0	acidic pH	(*RS*)-flurbiprofen, L-Ala–L-Ala	opaque hydrogel, poor stability at rt

The hydrogelation of **1a** also offers an opportunity to examine whether the attachment of a tyrosine phosphate residue to **1a** allows enzymatic hydrogelation. As a soluble precursor, **1c** (15.0 mg) dissolves in water (1.0 mL) at pH 7.6. After being treated with alkaline phosphatase (5.0 U/mL), the solution of **1c** quickly transforms to a clear hydrogel within one hour. Due to the additional tyrosine residue, **1d**, however, exhibits much better solubility in water than does **1a**. Thus, **1d** remains as a solution at the concentration of 0.8 wt %, and only forms a stable hydrogel (Figure 1C) when the concentration of **1d** reaches 1.5 wt %. As in the case of **1a**, the hydrogel of **1d** also shows good recovery properties.

Being the diastereomer of **1a**, hydrogelator **1b** exhibits quite different behavior in terms of self-assembly and hydrogelation. For example, at a concentration of 0.8 wt %, **1b** forms a solution at pH 9.0 and 60 °C, and the solution remains after the conditions have been adjusted to pH 7.0 and room temperature. Further decreasing of the pH value of the solution of **1b** to 4.0 results in an opaque hydrogel (Figure 1B), which lacks thermal reversibility and turns into white precipitates when it is heated to about 60 °C. Additionally, the white and opaque color of the hydrogel suggests that **1b** tends to form microcrystals [65–68] that constitute the weakly cross-linked matrices of the hydrogel. The addition of a D-tyrosine phosphate residue to **1b** affords the precursor **1e**. Despite containing a D-tyrosine phosphate, **1e** (0.8 wt %, pH 7.6) undergoes dephosphorylation in the presence of alkaline phosphatase (5.0 U/mL) to form the hydrogelator **1f**, which results in a transparent hydrogel (Figure 1D). Unlike **1d**, only 0.8 wt % of **1f** is necessary to form the hydrogel at pH 7.6.

As a hydrogelator, **2a** displays different characteristics to those of **2b**. **2a** (8.0 mg) dissolves in water (1.0 mL) at pH 9 when heated to 85 °C. The clear solution of **2a** turns into a stable, thermally reversible, and slightly opaque hydrogel (Figure 1E) after being stored at room temperature for 12 h at pH 7.2. **2b** exhibits a better solubility than **2a** because 0.8 wt % of **2b** gives a clear solution at pH 9.0 when being heating to 65 °C. Similarly, the solution of **2b** turns into a semitransparent hydrogel (Figure 1F) after the pH is decreased to 7.2. **2b** forms thermally reversible hydrogel within 2 min after its solution is cooled to room temperature from 65 °C, which is much faster than the hydrogelation of **2a**. The commercially available flurbiprofen is the racemic mixture **3**. Similar to the behavior of **2a**, **3a** (8.0 mg) dissolves in water (1.0 mL) at pH 9.0 and 75 °C, and the solution of **3a** becomes a stable semitransparent hydrogel (Figure 1G) within 5 min at room temperature after the pH of the solution is adjusted to 7.2. Although being thermally reversible, the hydrogel of **3a** exhibits poor recovery: the hydrogel of **3a** takes more than 24 hours to recover from the sol–gel state after being disrupted by an external force. Being made from a racemic mixture of ibuprofen (**4**), **4a** (8.0 mg) dissolves in water (1.0 mL) at pH 9 and 75 °C, and the solution of **4a** becomes a stable semitransparent hydrogel (Figure 1H) after standing at room temperature and pH 7.2 overnight. Despite its thermal reversibility, **4a** shows weak recovery properties. Compound **5a** fails to form a hydrogel, likely due to the insufficient aromatic–aromatic interaction originating from the salicylic acid groups. In order to confirm that the aromatic–aromatic interactions originating from Phe–Phe are essential for the self-assembly of the hydrogelators of NSAIDs to form the nanofibers as the matrices of the hydrogels, we studied the gelation properties of the conjugates of NSAIDs and Ala–Ala, a nonaromatic dipeptide. The conjugates of NSAIDs and Ala–Ala, **1g**, **3g**, **4g**, and **5g**, behave differently from those conjugates that contain Phe–Phe. At a concentration of 0.8 wt %, while **1g** and **3g** afford white, opaque hydrogels at pH 4.0 and 1.0, respectively, compounds **4g** and **5g** fail to form a hydrogel. The hydrogels of **1g** and **3g** shrink within 30 min after hydrogelation and tend to precipitate at room temperature over 24 h, indicating poor stability of the hydrogels of **1g** and **3g**. This result further confirms the critical role of aromatic–aromatic interaction for stabilizing molecular self-assembly in water.

### Transmission electron microscopy (TEM)

**Figure 2:**
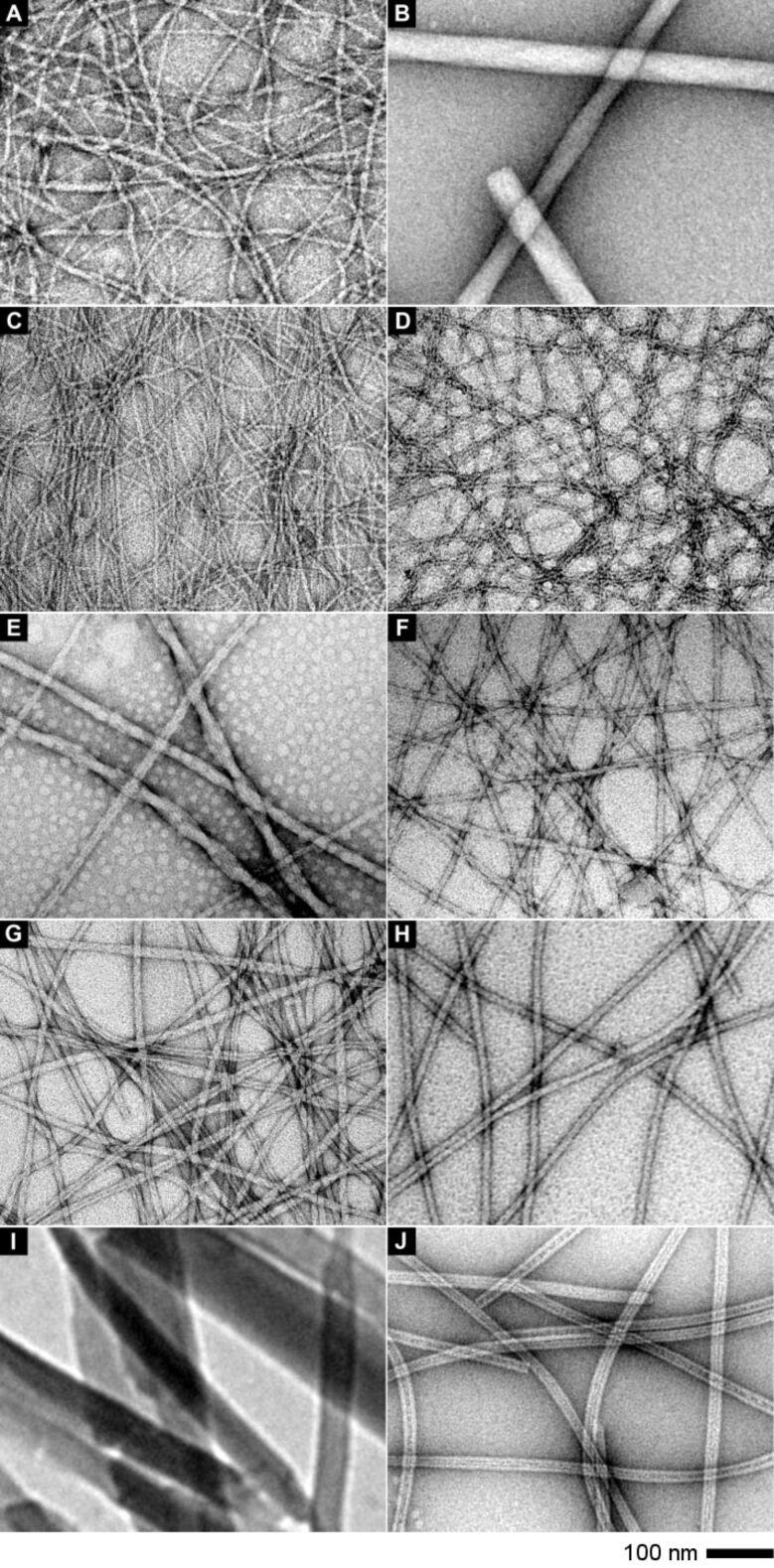
The TEM images of the matrices of the hydrogels formed by (A) **1a** (0.8 wt %, pH 7.0); (B) **1b** (0.8 wt %, pH 4.0); (C) **1d** (1.5 wt %, pH 7.6) formed by treating **1c** with alkaline phosphatase (5.0 U/mL); (D) **1f** (0.8 wt %, pH 7.6) formed by treating **1e** with alkaline phosphatase (5.0 U/mL); (E) **2a** (0.8 wt %, pH 7.2); (F) **2b** (0.8 wt %, pH 7.2); (G) **3a** (0.8 wt %, pH 7.2); (H) **4a** (0.8 wt %, pH 7.2); (I) **1g** (0.8 wt %, pH 4); (J) **3g** (0.8 wt %, pH 1.0). The scale bar is 100 nm.

In addition to the macroscopic phase transition such as hydrogelation, another hallmark of molecular self-assembly in water is the formation of ordered nanostructures (e.g., nanofibers or nanoparticles). As revealed by the TEM images (Figure 2), the hydrogel of **1a** comprises long nanofibers that entangle to form the network. The widths of nanofibers in the gel of **1a** appear to be nonuniform, displaying minimum width at 6 nm and maximum width at 20 nm (Figure 2A). The TEM image of the hydrogel of **1b** shows helical and rigid nanofibers with an average width of around 48 nm (Figure 2B), which is a different morphology from that of **1a,** and likely contributes to the thermal irreversibility of the hydrogel of **1b**. Besides, the low density of the network in hydrogel **1b** (Figure S1, Supporting Information File 1) [64] agrees with its poor reversibility. As shown in Figure 2C and Figure 2D, both the hydrogels of **1d** and **1f** comprise uniform long nanofibers, whose average widths are 6–7 nm. The exceptional high density of nanofibers in the hydrogel of **1d** likely originates from the relatively high concentration of **1d** in the hydrogel.

The hydrogel of **2a** comprises two kinds of nanostructures, i.e., nanofibers and nanoparticles, visible in the TEM images. As shown in Figure 2E, long nanofibers with an average width of 17 nm entangle together to form larger helical nanofibers with an average width of 30 nm. Higher-magnification TEM shows that the large helical fibers comprise fibrils of 17 nm in width (Figure S1, Supporting Information File 1) [64]. In addition to the presence of the nanofibers, the nanoparticles (10–20 nm in diameter) also exist in the hydrogel of **2a**. Different from the hydrogel of **2a**, hydrogel **2b** comprises long, uniform nanofibers with an average width of 12 nm (Figure 2F). Interestingly, the TEM of the hydrogel of **3a** presents similar nanostructures to those in the hydrogel of **2b**. As shown in Figure 2G, the long uniform nanofibers in the hydrogel of **3a** have an average width of 12 nm. Higher magnification of the TEM of the hydrogel of **3a** indicates that the nanofibers of **3a** consist of two nanofibrils with an average width of 6 nm (Figure S1, Supporting Information File 1) [64]. As shown in Figure 2H, the hydrogel of **4a** also consists of uniform nanofibers with an average width of 13 nm. These nanofibers arrange into a network that has very low density. While the TEM image (Figure 2I) of the hydrogel of **1g** clearly shows nanocrystals with a length over 450 nm and width varying from 35 nm to 200 nm, the TEM image (Figure 2J) of the hydrogel of **3g** exhibits uniform nanotubes that have an outer width of 22 nm and inner width of 8 nm. These nanotubes form a poorly cross-linked network, which agrees with the instability of the hydrogel of **3g**.

### Rheology

Since viscoelasticity is an essential feature of hydrogels [69], we use rheometry to characterize the hydrogels. Based on the stability of these hydrogels, we choose to study the rheological properties of the hydrogels of **1a**, **1b**, **1d**, **1f**, **2a**, **2b**, **3a** and **4a**, and list their rheology data in Table 2. During the dynamic strain sweep, all these hydrogels, under constant oscillation frequencies and different oscillation strains, exhibit a strain-independent storage modulus (*G'*) until reaching their critical strain, at which the values of *G′* start to decrease drastically due to the breakdown of the networks of the hydrogels. In addition to the critical strain, the dynamic strain sweep provides the maximum *G'* values in the linear region. The naproxen-containing hydrogels show maximum storage moduli from 4.0 × 10^2^ to 4.9 × 10^4^ Pa (Table 2). The hydrogel of **1a** has a maximum *G'* of 2.1 × 10^3^ Pa and critical strain of 0.62%, and the hydrogel of **1b** has the highest maximum *G'* (4.9 × 10^4^ Pa), agreeing with the well cross-linked network in the hydrogel of **1b** resulted from the long and wide (48 nm) nanofibers. Since the tyrosine residue renders **1d** and **1f** with better solubility than for **1a** and **1b** (Table S1, Supporting Information File 1) [64], the hydrogels of **1d** and **1f** have smaller maximum storage moduli (6.2 × 10^2^ and 4.0 × 10^2^ Pa) than do the hydrogels of **1a** and **1b**. The thin and flexible nanofibers in the hydrogels of **1d** and **1f** also agree with their rheological properties.

**Table 2:** The rheological properties and TEM characteristics of the NSAID-containing hydrogels.

compound	cgc (wt %)	max. *G'* ^a^ (Pa)	critical strain^a^ (%)	*G'* ^b^ (Pa)	nanofiber width^c^ (nm)

**1a**	0.2	2100	0.62	1700	6–20
**1b**	0.2	49000	1.0	53000	48
**1d**^d^	1.0	620	0.39	610	6.5
**1f**	0.2	400	1.6	620	7
**2a**	0.2	150	0.96	150	17
**2b**	0.2	890	1.8	990	12
**3a**	0.2	1300	3.7	1500	6
**4a**	0.3	7.8	0.57	13	13

^a^Dynamic strain sweep; ^b^Dynamic frequency sweep, the value is taken at a frequency equal to 6.28 rad/s; ^c^Average; ^d^The concentration of hydrogel **1d** is 1.5 wt %, while the others are 0.8 wt %.

(*R*)-Flurbiprofen-containing hydrogelators **2a** and **2b** exhibit maximum storage moduli of 1.5 × 10^2^ and 8.7 × 10^2^ Pa, respectively, which are smaller than those of the hydrogels of **1a** and **1b**. Such a difference is consistent with the less aromatic–aromatic interaction provided by the flurbiprofen motif due to its smaller conjugate system compared with the naproxen group in **1a** and **1b**. The maximum *G'* of the hydrogel of **2b** (8.9 × 10^2^ Pa) is larger than that of the hydrogel of **2a** (1.5 × 10^2^ Pa), agreeing with the higher density of the nanofibers in the hydrogel of **2b**. Interestingly, the gel of **3a**, which comprises **2a** and (*S*)-flurbiprofen-FF, exhibits a higher maximum *G'* (1.3 × 10^3^ Pa) in strain sweep and larger critical strain (3.7%) than those values of **2a** or **2b**, suggesting that the combination of **2a** and (*S*)-flurbiprofen-FF affords a more stable hydrogel. This result agrees with the TEM image of **3a**, which exhibits the highest density of its network among the flurbiprofen-containing hydrogels. Unlike the hydrogels containing naproxen or flurbiprofen, ibuprofen-containing hydrogel **4a** behaves as a relatively weak hydrogel and exhibits a maximum *G'* of 7.8 Pa and critical strain 0.57%, clearly indicating that the isopropyl-substituted phenyl group is less efficient at providing aromatic–aromatic interaction for hydrogelation than naproxen or flubiprofen group are.

After obtaining the critical strains of the hydrogels, we measure the frequency dependence of their storage and loss moduli using dynamic frequency sweep at constant oscillation amplitude and temperature but varying oscillation frequency (0.1–200 rad/s). All of these hydrogels exhibit little dependence on frequency (Figure S3, Supporting Information File 1) [64], suggesting that the matrices of gels have good tolerance to the external shear force. In addition, the storage moduli of all these hydrogels in the frequency sweep are comparable with storage moduli of the constant region in the strain sweep. Hydrogel **1b** has the highest *G'* (5.3 × 10^4^ Pa), while hydrogels **1d** and **1f** appear to have the lowest two *G'* values (6.1 × 10^2^ Pa and 6.2 × 10^2^ Pa) among the naproxen-containing hydrogels. Hydrogels of **2a** and **2b** have smaller *G'* values, 1.5 × 10^2^ Pa and 9.9 × 10^2^ Pa, respectively, than those of hydrogels **1a** and **1b**, which likely originate from their structure difference. Hydrogel **3a** has a *G'* value of 1.5 × 10^3^ Pa, which is larger than that of hydrogel of **4a** (13 Pa), partially due to more effective aromatic–aromatic interaction of the flurbiprofen groups compared to the ibuprofen.

**Figure 3:**
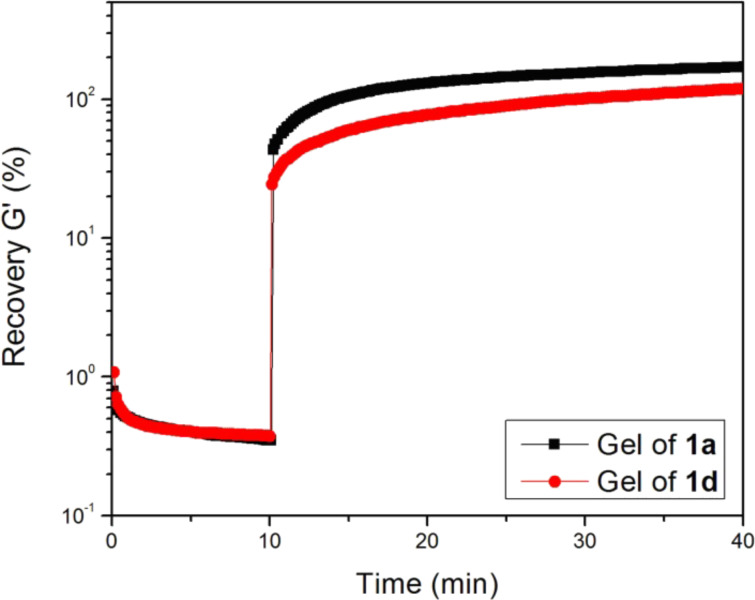
Recovery of the storage moduli of the gels formed by 0.8 wt % of **1a** at pH 7.0 and 1.5 wt % of **1d** formed by using alkaline phosphatase (5.0 U/mL) to treat the solution of **1c** at pH 7.6.

Both hydrogels of **1a** and **1d** possess excellent thermal recovery properties (Figure S4, Supporting Information File 1) [64], indicating that these molecules self-assemble to re-establish the network rapidly after deformation caused by a perturbation. To characterize the recovery properties of the gels of **1a** and **1d**, we first measure the *G'* values of the gels at 0.4% strain for 10 min to obtain their original storage moduli, and then apply a large-amplitude oscillation with 100% strain and 6.28 rad/s angular frequency to perturb the structure of the hydrogels for 10 min. After removing this large amplitude of oscillation, we immediately measure the storage moduli of the gels at 0.4% of strain, and then calculate their recovery percentages by dividing them by their original moduli. As shown in Figure 3, *G'* of hydrogel **1a** decreases to 0.5% of its original storage modulus when being exposed to the large-amplitude oscillation (100% of strain). After the removal of the applied large oscillation, the *G'* value of hydrogel **1a** immediately recovers by 50% and reaches 100% recovery in less than 5 min. Similarly, hydrogel **1d** also exhibits a fairly fast recovery of *G'*. The application of the large oscillation for 10 min decreases the *G'* of hydrogel **1d** to 2.5% of its original storage modulus. As soon as the external large oscillation is stopped, hydrogel **1d** recovers by 25% and reaches 100% recovery after 20 min. These data suggest that hydrogels **1a** and **1d**, like other supramolecular gels [70–71], are able to re-establish networks rapidly after deformation.

### Cytotoxicity

**Figure 4:**
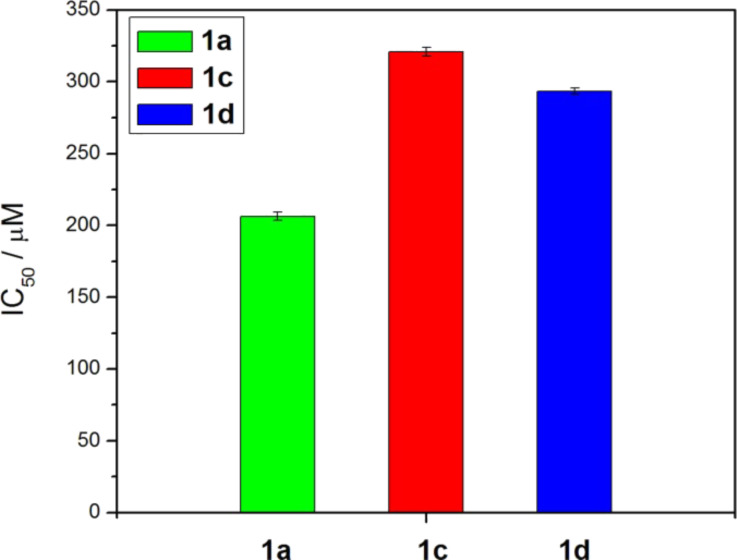
The IC_50_ values of **1a**, **1c** and **1d** incubated with HeLa cells after 72 h.

To evaluate the biocompatibility of these NSAID hydrogelators, we select **1a**, **1c**, and **1d** to test their cytotoxicity by incubating them with HeLa cells for 72 h at 37 °C. As shown in Figure 4, **1a**, **1c**, and **1d** exhibit IC_50_ values of 206 µM, 321 µM, and 294 µM, respectively. Among these three hydrogels, **1a** lacks the tyrosine residue and is less soluble in water, thus exhibiting relatively low IC_50_. All of **1a**, **1c** and **1d**, exhibit IC_50_ values larger than 200 µM, suggesting that they are relatively biocompatible.

## Conclusion

In conclusion, we have systematically investigated a new type of supramolecular hydrogelator made of nonsteroidal anti-inflammatory drugs (NSAIDs). The direct conjugation of NSAIDs and small peptides (Phe–Phe) generates molecules that self-assemble in water to form hydrogels. The large difference in gelation properties by changing small peptides from Phe–Phe to Ala–Ala confirms the important role of the aromatic–aromatic interaction between diphenylalanine groups [24]. In addition, the molecular structures of NSAIDs also affect their gelation stability. As a useful member in NSAIDs, naproxen conjugates with Phe–Phe to give a promising hydrogelator (NpxFF) that may act as a general motif to enable hydrogelation of other bioactive molecules. By providing useful insight for design of the NSAID hydrogelators, this approach contributes to the development of bioactive molecules that have dual or multiple roles, such as hydrogelators and therapeutic agents.

## Supporting Information

File 1 Experimental details.
