# Supplementary material for: The conjugation of nonsteroidal anti-inflammatory drugs (NSAID) to small peptides for generating multifunctional supramolecular nanofibers/hydrogels
Source: Beilstein J Org Chem. 2013 May 10;9:908–17. doi: 10.3762/bjoc.9.104 (PMC3678635; doi:10.3762/bjoc.9.104)
Supplement: File 1 — Experimental details. [file Beilstein_J_Org_Chem-09-908-s001.pdf]

Supporting Information

for

The conjugation of nonsteroidal anti-inflammatory drugs (NSAID) to small peptides for generating multifunctional supramolecular nanofibers/hydrogels

Jiayang Li, Yi Kuang, Junfeng Shi, Yuan Gao, Jie Zhou and Bing Xu*

Address: Department of Chemistry, Brandeis University, 415 South Street, Waltham, MA 02454, USA

Email: Bing Xu* - bxu@brandeis.edu

* Corresponding author

Experimental details

General methods and materials. All of the chemical reagents and solvents were used as received from the commercial sources without further purification unless otherwise noted. Flash chromatography was performed on silica gel 60 (230-400 mesh). Analytical thin layer chromatography (TLC) was performed using silica gel 60 F_{254} pre-coated glass plates (0.25 mm) and analyzed by short wave UV illumination. ^{1}H and ^{31}P NMR spectra were obtained on Varian Unity Inova 400. Chemical shifts are reported in δ (ppm) relative to the solvent residual peak (phosphoric acid for ^{31}P NMR). Coupling constants are reported in Hz with multiplicities denoted as s (singlet), d (doublet), t (triplet), q (quartet), p (pentet), m (multiplet) and br (broad). The Hela cell line (CCL2) was purchased from American Type Culture Collection. All of the culture media were provided from Invitrogen. COX inhibitor screening assay kit (700100) was purchased form Cayman Chemical Company. Cytotoxicity test and COX inhibition tests were measured by DTX 880 multimode detector. Rheological data were measured on ARES G2 rheometer. TEM images were taken on a Morgagni 268 transmission electron microscope.

Naproxen–L-Phe–L-Phe (1a). 690 mg (3 mmol) of naproxen (**1**) was reacted with 345 mg (3 mmol) of *N*-hydroxysuccinimide (NHS) and 680 mg (3.3 mmol) of *N,N'*-dicyclohexylcarbodiimide (DCC) in chloroform for 2 h at room temperature. Filtration, evaporation, and recrystallization in ethanol gave pure naproxen-NHS ester. A solution of the pure naproxen-NHS ester in acetone was added to an aqueous solution (pH was adjusted to 8.5 by Na_2CO_3) of 558 mg (3 mmol) of L-phenylalanine, and the resulting solution was stirred for 12 h at room temperature. The solution was concentrated by rotary evaporator until all the acetone was removed. 1 M of HCl was added to adjust the pH of the remaining aqueous solution to 3.0 and the resulting white precipitate was collected by filtration. Flash chromatography of the crude product on silica gel gave pure naproxen-F (**11**). The same coupling procedure was repeated to afford 976 mg (62% from **1**) of naproxen-FF (**1a**) as a white solid: ^{1}H NMR (400 MHz, DMSO-d_6) 8.27 (d, *J* = 4.1 Hz, 1H), 8.14 (d, *J* = 7.9 Hz, 1H), 7.80-7.57 (m, 3H), 7.33 (d, *J* = 8.0 Hz, 1H), 7.29-6.97 (m, 12H), 4.59 (t, *J* = 12.1 Hz, 1H), 4.36 (q, *J* = 7.9 Hz, 1H), 3.85 (s, 3H), 3.72 (q, *J* = 8.0 Hz, 1H), 3.08-2.65 (m, 4.1H), 1.18 (d, *J* = 8.1 Hz, 3H).

Naproxen–L-Phe–L-Phe–L-Tyr phosphate (1c). 524 mg (1 mmol) of **1a** was reacted with 115 mg (1 mmol) of NHS and 227 mg (1.1 mmol) of DCC in chloroform for 12 h at room temperature. The crude product of naproxen-FF-NHS ester was collected by filtration and evaporation and purified by recrystallization in hexane and ethanol (10:1). A solution of the pure naproxen-FF-NHS in acetone was added to a solution (pH was adjusted to 8.5 by 210 mg (2 mmol) of Na_2CO_3) of 261 mg (1 mmol) of L-tyrosine phosphate and in DI water, and the resulting solution was stirred for 24 h at room temperature. The solution was then concentrated by rotary evaporator until all the acetone was removed. 1 M of HCl was added to adjust the pH of the remaining aqueous solution to 1.0 and the resulting white precipitate was collected by filtration. Purification with HPLC gave 346 mg (45% from **1**) of pure naproxen-FFY(p) (**1c**) as a white solid: ^{1}H NMR (400 MHz, DMSO-d_6) 8.30 (s, 1H), 8.11 (m, 1H), 7.78-7.56 (m, 3H), 7.39-6.95 (m, 17H), 4.51 (s, 2H), 4.41 (s, 1H), 3.84 (s, 3H), 3.72 (s, 1H), 3.08-2.62 (m, 4H), 1.17 (s, 3H); ^{31}P NMR (160 MHz, DMSO-d_6) −5.74.

Naproxen–D-Phe–D-Phe (1b). The same procedure as for **1a** was applied, using 692 mg (3 mmol) of naproxen (**1**) and 560 mg (3 mmol) of D-phenylalanine. After flash chromatography, 1169 mg (74% from **1**) of naproxen-ff (**1b**) as a white solid: ^{1}H NMR (400 MHz, DMSO-d_6) 8.32 (d, *J* = 8.0 Hz, 1H), 8.08 (d, *J* = 8.1 Hz, 1H), 7.71 (d, *J* = 8.1 Hz, 1H), 7.62 (d, *J* = 7.9 Hz, 1H), 7.56 (s, 1H), 7.38-7.07 (m, 9H), 7.02-6.85 (m, 4H), 4.56-4.38 (m, 2H), 3.86 (s, 3H), 3.76 (q, *J* = 8.0 Hz, 1H), 3.13-2.60 (m, 4H), 1.32 (d, *J* = 8.0 Hz, 3H).

Naproxen–D-Phe–D-Phe–D-Tyr phosphate (1e). The same procedure as for **1a** was applied, using 1.05 g (2 mmol) of **1b** and (2 mmol) of D-tyrosine phosphate. Purification with HPLC gave 737 mg (48% from **1b**) of pure naproxen-ffy(p) (**1e**) as a white solid: ^{1}H NMR (400 MHz, DMSO-d_6) 8.38 (s, 1H), 8.14 (s, 1H), 8.05 (s, 1H), 7.70 (d, *J* = 8.0 Hz, 1H), 7.60 (d, *J* = 8.1 Hz, 1H), 7.56 (s, 1H), 7.37-7.02 (m, 12H), 7.00-6.78 (m, 5H), 4.58 (s, 1H), 4.44 (s, 2H), 3.86 (s, 3H), 3.76 (s, 1H), 3.13-2.56 (m, 4H), 1.30 (s, 3H); ^{31}P NMR (160 MHz, DMSO-d_6) −5.69.

Naproxen–L-Ala–L-Ala (1g). 231 mg (1 mmol) of naproxen (**1**) was reacted with 115 mg (1 mmol) of NHS and 227 mg (1.1 mmol) of DCC in chloroform for 2 h at room temperature. Filtration, evaporation, and recrystallization in ethanol (6 mL) gave pure naproxen-NHS ester. A solution of the pure naproxen-NHS ester in acetone was added to a solution (pH was adjusted to 8.5 by Na_2CO_3) of 162 mg (1 mmol) of this L-alanine-L-alanine, which was prepared by solid phase peptide synthesis (2-chlorotrityl chloride resin, 100–200 mesh and 0.3–0.8 mmol/g), in 5 mL of DI water, and the resulting solution was stirred for 24 h at room temperature. The solution was then concentrated by rotary evaporator until all the acetone was removed. 1 M of HCl was added to adjust the pH of the remaining aqueous solution to 3.0 and the resulting white precipitate was collected by filtration. Flash chromatography on silica gel gave 283 mg (76% from **1**) of pure naproxen-AA (**1g**) as a white solid: ^{1}H NMR (400 MHz, $CDCl_3$) 7.78-7.59 (m, 2H), 7.35 (d, J = 7.9 Hz, 1H), 7.15 (d, J = 8.0 Hz, 1H), 7.11 (s, 1H), 6.77 (d, J = 8.1 Hz, 1H), 6.05 (d, J = 7.9 Hz, 1H), 4.54 (t, J = 8.0 Hz, 1H), 4.39 (t, J = 8.0 Hz, 1H), 3.91 (s, 3H), 3.72 (q, J = 8.0 Hz, 1H), 1.59 (d, J = 8.1 Hz, 3H), 1.29 (d, J = 3.9 Hz, 3H), 1.25 (d, J = 3.9 Hz, 3H).

(*R*)-Flurbiprofen–L-Phe–L-Phe (2a). 244 mg (1 mmol) of (*R*)-flurbiprofen (**2**) was reacted with 115 mg (1 mmol) of NHS and 227 mg (1.1 mmol) of DCC in chloroform for 3 h at room temperature. Filtration, evaporation, and flash chromatography gave pure (*R*)-flurbiprofen-NHS ester. A solution of the pure naproxen-NHS ester in acetone was added to a solution (pH was adjusted to 8.5 by Na_2CO_3) of 313 mg (1 mmol) of L-phenylalanine–L-phenylalanine (**6**), which was prepared by solid phase peptide synthesis (2-chlorotrityl chloride resin), in 8 mL of DI water, and the resulting solution was stirred for 24 hours at room temperature. The solution was then concentrated by rotary evaporator until all the acetone was removed. 1 M of HCl was added to adjust the pH of the remaining aqueous solution to 3.0 and the resulting white precipitate was collected by filtration. Purification with HPLC gave 172 mg (32% from **2**) of pure (*R*)-flurbiprofen-FF (**2a**) as a white solid: ^{1}H NMR (400 MHz, $CDCl_3$) 7.55-6.81 (m, 18H), 6.76 (d, J = 8.0 Hz, 1H), 6.24 (d, J = 8.1 Hz, 1H), 4.73 (q, J = 8.1 Hz, 2H), 3.43 (q, J = 8.0 Hz, 1H), 3.91-3.10 (m, 1H), 3.01-2.80 (m, 3H), 1.39 (d, J = 7.9 Hz, 3H).

(*R*)-Flurbiprofen–D-Phe–D-Phe (2b). 244 mg (1 mmol) of (*R*)-flurbiprofen (**2**) was reacted with 115 mg (1 mmol) of NHS and 227 mg (1.1 mmol) of DCC in chloroform for 3 h at room temperature. Filtration, evaporation, and flash chromatography gave pure (*R*)-flurbiprofen-NHS ester. A solution of the pure naproxen-NHS ester in acetone was added to a solution (pH was adjusted to 8.5 by Na_2CO_3) of 313 mg (1 mmol) of D-phenylalanine–D-phenylalanine (**7**), which was prepared by solid phase peptide synthesis (2-chlorotrityl chloride resin), in 8 mL of DI water, and the resulting solution was stirred for 24 h at room temperature. The solution was then concentrated by rotary evaporator until all the acetone was removed. 1 M of HCl was added to adjust the pH of the remaining aqueous solution to 3.0 and the resulting white precipitate was collected by filtration. Purification with HPLC gave 220 mg (41% from **2**) of pure (*R*)-flurbiprofen-ff (**2b**) as a white solid: ^{1}H NMR (400 MHz, $CDCl_3$) 7.61-6.84 (m, 18H), 6.43 (d, J = 8.0 Hz, 1H), 6.17 (d, J = 8.1 Hz, 1H), 4.68 (q, J = 8.2 Hz, 2H), 3.53 (q, J = 8.0 Hz, 1H), 3.10-2.83 (m, 4H), 1.41 (d, J = 8.1 Hz, 3H).

(*RS*)-Flurbiprofen-L-Phe-L-Phe (3a). Solid phase peptide synthesis with 1000 mg of 2-chlorotrityl chloride resin (100~200 mesh and 0.3~0.8 mmol/g), 490 mg (2 mmol) of (RS)-flurbiprofen (**3**) and 1548 mg (4 mmol) of Fmoc-L-phenylalanine and purification with HPLC gave 371 mg (86% of resin) of a 1:1 mixture of (R)-flurbiprofen-FF and (S)-flurbiprofen-FF as a white solid: ^{1}H NMR (400 MHz, DMSO-d_6) 8.36 (m, 1H), 8.22 (m, 1H), 7.60-6.94 (m, 18H), 4.65-4.32 (m, 2H), 3.69 (s, 1H), 3.16-2.81 (m, 3H), 2.79-2.58 (m, 1H), 1.25 (d, J = 7.9 Hz, 1.5H), 1.12 (d, J = 7.9 Hz, 1.5H).

(*RS*)-Flurbiprofen–L-Ala–L-Ala (3g). Solid phase peptide synthesis with 1000 mg of 2-chlorotrityl chloride resin (100–200 mesh and 0.3–0.8 mmol/g), 490 mg (2 mmol) of (*RS*)-flurbiprofen (**3**) and 1245 mg (4 mmol) of Fmoc-L-alanine and purification with HPLC gave 281 mg (91% of resin) of a 1:1 mixture of (*R*)-flurbiprofen-AA and (*S*)-flurbiprofen-AA as a white solid: ^{1}H NMR (400 MHz, DMSO-d_6) 8.27 (s, 1H), 8.20 (s, 0.5H), 8.10 (s, 0.5H), 7.57-7.31 (m, 6H), 7.30-7.26 (m, 2H), 4.40-4.06 (m, 2H), 3.79 (s, 1H), 1.34 (s, 3H), 1.28 (d, J = 8.0 Hz, 1.5H), 1.21 (s, 3H), 1.15 (d, J = 8.1 Hz, 1.5H).

(*RS*)-Ibuprofen–L-Phe–L-Phe (4a). Solid phase peptide synthesis with 1000 mg of 2-chlorotrityl chloride resin (100–200 mesh and 0.3–0.8 mmol/g), 412 mg (2 mmol) of (*RS*)-ibuprofen (**4**) and 1548 mg (4 mmol) of Fmoc-L-phenylalanine and purification with HPLC gave 312 mg (78% of resin) of a 1:1 mixture of (*R*)-ibuprofen-FF and (*S*)-ibuprofen-FF as a white solid: ^{1}H NMR (400 MHz, DMSO-d_6) 8.28 (t, J = 7.9 Hz, 1H), 8.07 (d, J = 8.2 Hz, 0.5H), 8.01 (d, J = 8.0 Hz, 0.5H), 7.32-6.92 (m, 14H), 4.63-4.32 (m, 2H), 3.66-3.49 (m, 1H), 3.14-2.80 (m, 3H), 2.78-2.62 (m, 1H), 2.38 (d, J = 4.1 Hz, 2H), 1.78 (m, 1H), 1.21 (d, J = 8.0 Hz, 1.5H), 1.06 (d, J = 8.1 Hz, 1.5H), 0.84 (t, J = 5.8 Hz, 6H).

(*RS*)-Ibuprofen–L-Ala–L-Ala (4g). Solid phase peptide synthesis with 1000 mg of 2-chlorotrityl chloride resin (100–200 mesh and 0.3–0.8 mmol/g), 412 mg (2 mmol) of (*RS*)-ibuprofen (**4**) and 1245 mg (4 mmol) of Fmoc-L-alanine and purification by HPLC gave 245 mg (88%) of a 1:1 mixture of (*R*)-ibuprofen-AA and (*S*)-ibuprofen-AA as a white solid: ^{1}H NMR (400 MHz, $CDCl_3$) 7.22-7.02 (m, 4H), 6.98 (d, J = 8.0 Hz, 0.5H), 6.91 (d, J = 7.9 Hz, 0.5H), 6.08 (t, J = 7.9 Hz, 1H), 4.51-4.37 (m, 2H), 3.56 (q, J = 8.0 Hz, 1H), 2.44 (q, J = 11.9 Hz, 2H), 1.84 (m, 1H), 1.49 (d, J = 4.0 Hz, 3H), 1.42 (d, J = 8.1 Hz, 1.5H), 1.30 (d, J = 4.0 Hz, 3H), 1.25 (d, J = 8.1 Hz, 1.5H), 0.88 (d, J = 8.0 Hz, 3H).

Salicylic acid–L-Phe–L-Phe (5a). 181 mg (1 mmol) of aspirin (**5**) was reacted with 115 mg (1 mmol) of NHS and 226 mg (1.1 mmol) of DCC in chloroform for 3 h at room temperature. Filtration, evaporation, and flash chromatography gave pure aspirin-NHS ester. A solution of the pure naproxen-NHS ester in 5 mL of acetone was added to a solution of 313 mg (1 mmol) of L-phenylalanine–L-phenylalanine (**6**), which was prepared by solid phase peptide synthesis (2-chlorotrityl chloride resin), and 105 mg (1 mmol) of Na_2CO_3 in 8 mL of DMF, and the resulting solution was stirred for 24 h at room temperature. The solution was concentrated by rotary evaporator. Purification of the residue with HPLC gave 242 mg (56% from **5**) of pure salicylic acid-FF (**5a**) (aspirin was hydrolyzed during the synthesis) as a white solid: ^{1}H NMR (400 MHz, DMSO-d_6) 8.85 (d, J = 8.0 Hz, 1H), 8.52 (d, J = 4.1 Hz, 0.5H), 7.87 (d, J = 8.0 Hz, 0.5H), 7.56-7.00 (m, 10H), 6.87 (s, 1H), 4.79 (s, 1H), 4.47 (s, 1H), 3.20-2.84 (m, 4H).

Salicylic acid-L-Ala-L-Ala (5g). 181 mg (1 mmol) of aspirin (**5**) was reacted with 115 mg (1 mmol) of NHS and 226 mg (1.1 mmol) of DCC in chloroform for 3 h at room temperature. Filtration, evaporation, and flash chromatography gave pure aspirin-NHS ester. A solution of the pure naproxen-NHS ester in 5 mL of acetone was added to a solution of 162 mg (1 mmol) of L-alanine–L-alanine (**10**), which was prepared by solid phase peptide synthesis (2-chlorotrityl chloride resin), and 105 mg (1 mmol) of Na_2CO_3 in 5 mL of DMF, and the resulting solution was stirred for 24 h at room temperature. The solution was concentrated by rotary evaporator. Purification of the residue with HPLC gave 170 mg (61% from **5**) of pure salicylic acid-AA (**5g**) (aspirin was hydrolyzed during the synthesis) as a white solid: ^{1}H NMR (400 MHz, $CDCl_3$) δ 7.53 (t, *J* = 8.0 Hz, 1H), 7.40 (t, *J* = 8.1 Hz, 1H), 6.97 (d, *J* = 8.0 Hz, 0.5H), 6.86 (t, *J* = 8.1 Hz, 1H), 4.75 (d, *J* = 7.9 Hz, 1H), 4.59 (t, *J* = 7.9 Hz, 1H), 1.67-1.31 (m, 6H).

Cytotoxicity. 5×10^5 (cells/well) of health HeLa cells were seeded into 96-well plates with 100 μL of MEM medium supplemented with 10% fetal bovine serum (FBS), 100 U/mL penicillin and 100 mg ml21 streptomycin. Then the HeLa cells were incubated at 37 °C and 5% CO_2 for 12 h in an incubator, which allowed cells to attach to the bottom of 96-well plates. Then the medium was replaced by another 100 μL of growth medium that contained serial diluents of our compounds (0.5% DMSO) for each well and the cells were incubated with our compound-diluted medium at 37 °C and 5% CO_2 for an additional 72 h. During the measurement of proliferation of HeLa cells, which were assayed into three days, 10 μL of (3-(4,5-dimethylthiazol-2-yl)-2,5-diphenyl-tetrazolium bromide (MTT, 0.5 mg/mL) was added into the assigned wells for their corresponding days every 24 h, followed by adding 100 μL of 0.1% sodium dodecyl sulfate (SDS) 4 h later. Then we collected the assay results after another incubation for 24 h. Since the mitochondrial reductase in living cells reduced MTT to purple fomazan, the absorbance at 595 nm of the whole solution was finally measured by DTX 880 Multimode Detector. With MEM medium as blank and untreated HeLa cells as control, we measure each concentration of these compounds in triplicate. The IC50 values of our hydrogelators were read from their activity curves (with the measurement of 5 different concentrations) at day 3.

Critical gelation concentration of NSAID containing hydrogels. To measure the critical gelation concentration (cgc) of NSAID containing hydrogels, we prepared hydrogels with concentrations of 1.0 wt %, 2.0 wt %, 4.0 wt %, 6.0 wt %, 8.0 wt % and 1.0 wt % respectively. After adjusting the conditions of solutions to their gelation conditions, which were described in our article, we kept the solutions at room temperature for 48 h and waited for them to form stable hydrogels. The results were listed in Table S1 [1].

Table S1: The critical gelation concentration (cgc) of the NSAID containing hydrogels and their solubility in water at pH 7.0 at room temperature.

	cgc (wt %)	solubility (g/100g)	optical image of the gel at cgc
**1a**	0.2	0.0553	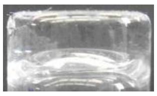
**1b**	0.2	0.0896	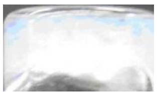
**1d**	1.0	0.433	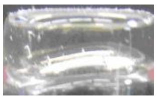
**1f**	0.2	0.247	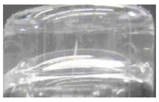
**2a**	0.2	0.0163	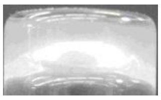
**2b**[a]	0.2	0.0308	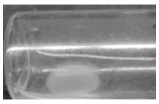
**3a**	0.2	0.0260	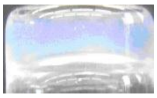
**4a**	0.3	0.0746	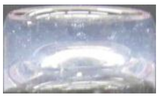
**1g**	0.3	0.305	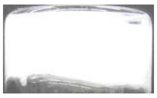
**3g**	0.8	0.188	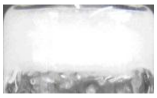

[a]The gel may form and shrink within 10 minutes.

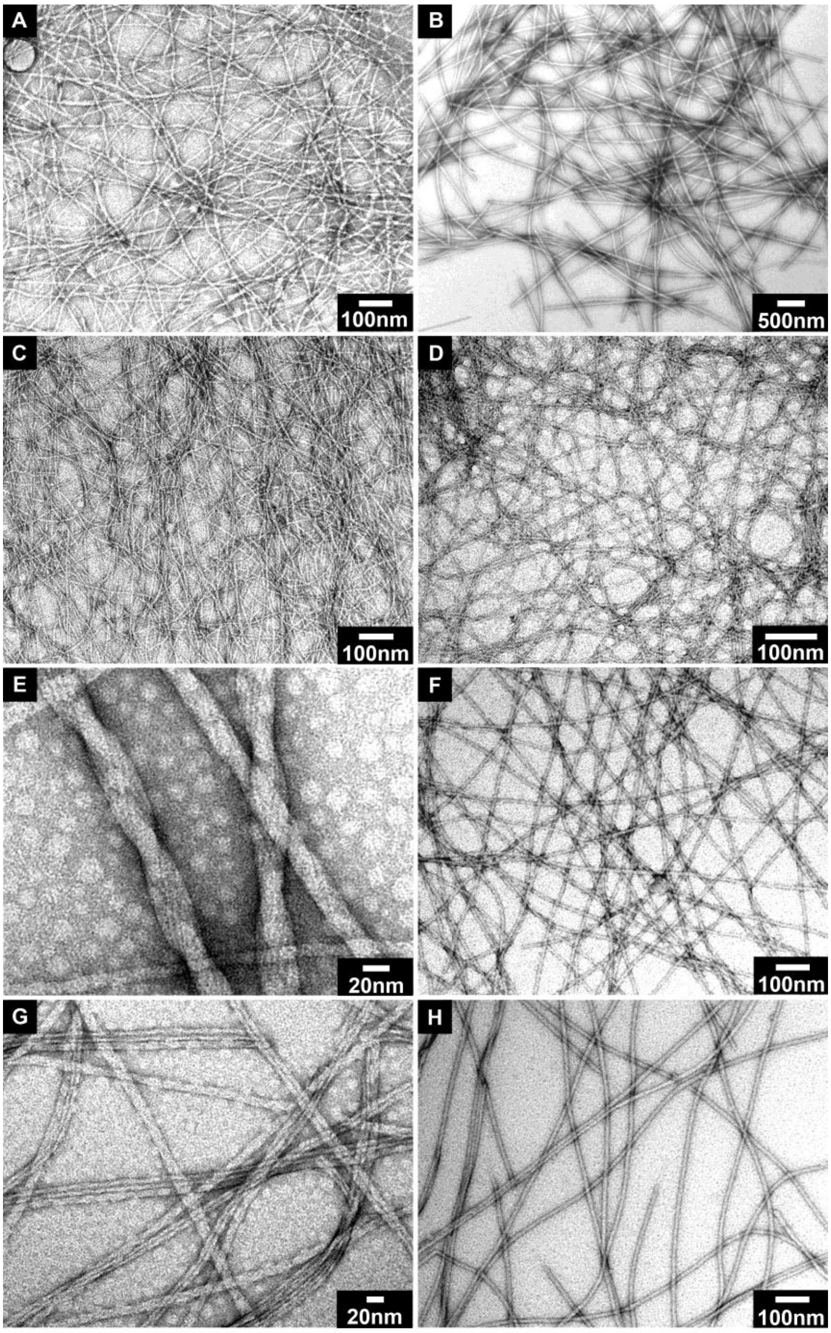

Figure S1: The TEM images of Phe–Phe and Phe–Phe–Tyr conjugated NSAID containing hydrogels: (A) the gel of **1a** (0.8 wt %, pH 7.0); (B) the gel of **1b** (0.8 wt %, pH 4.0); (C) the gel of **1d** (1.5 wt %, pH 7.6, 5 U/mL enzyme); (D) the gel of **1f** (0.8 wt %, pH 7.6, 5 U/mL enzyme); (E) the gel of **2a** (0.8 wt %, pH 7.2); (F) the gel of **2b** (0.8 wt %, pH 7.2); (G) the gel of **3a** (0.8 wt %, pH 7.2); (H) the gel of **4a** (0.8 wt %, pH 7.2).

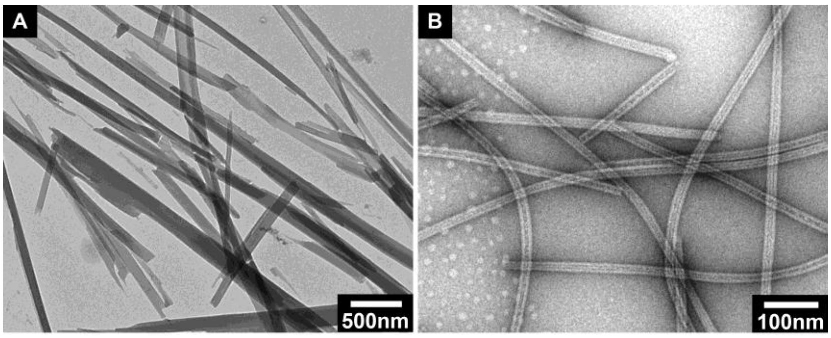

Figure S2: The TEM images of Ala–Ala conjugated NSAID containing hydrogels: (A) the gel of **1g** (0.8 wt %, pH 4.0); (B) the gel of **3g** (0.8 wt %, pH 1.0);

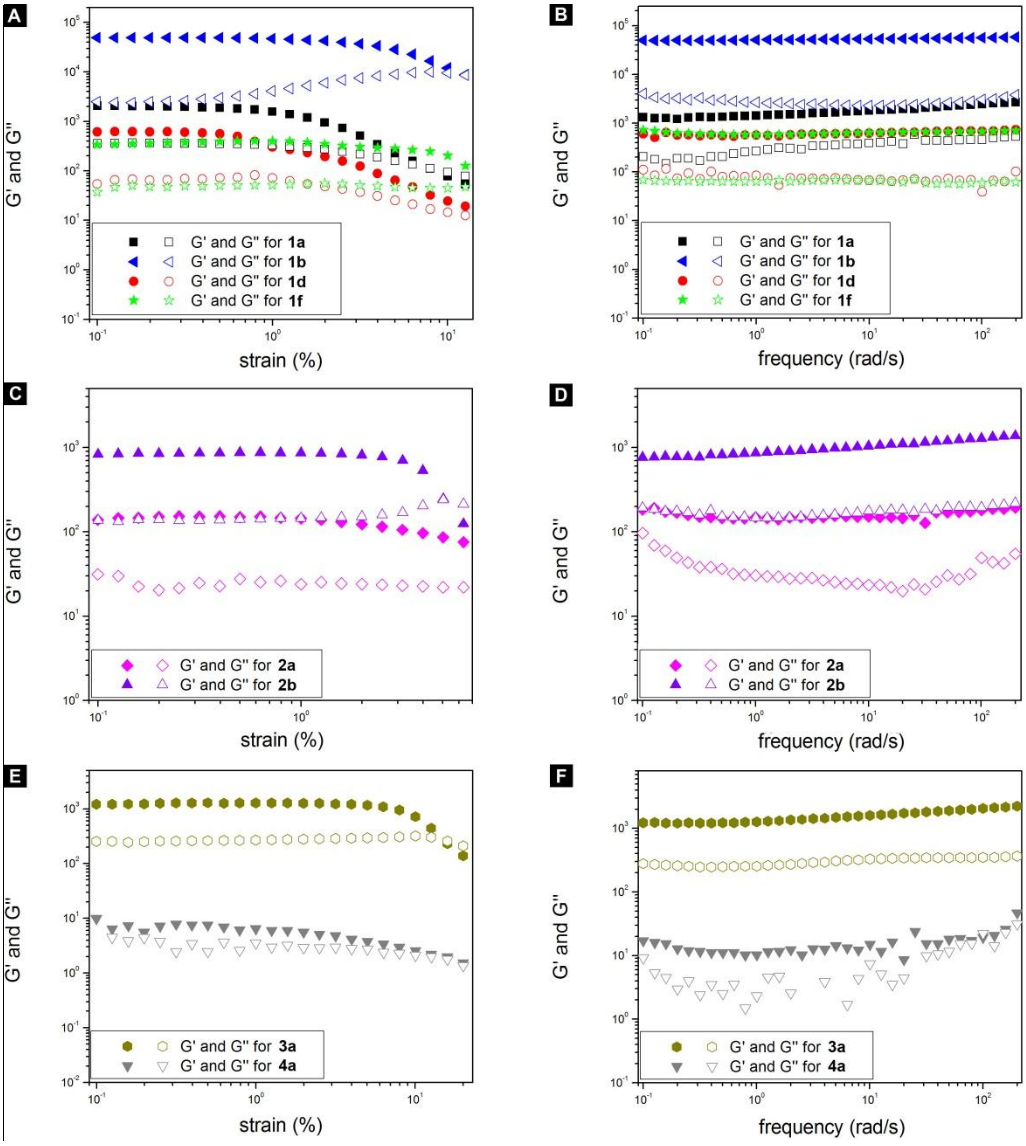

Figure S3: The strain (A, C, E) and frequency (B, D, F) dependence of the dynamic storage modulus G′ (solid) and loss modulus G″ (hollow) of naproxen based hydrogels (**1a**, **1b**, **1d** and **1f**), (*R*)-flurbiprofen based hydrogels (**2a** and **2b**), (*RS*)-flurbiprofen based hydrogel (**3a**) and (*RS*)-ibuprofen based hydrogels (**4a**).

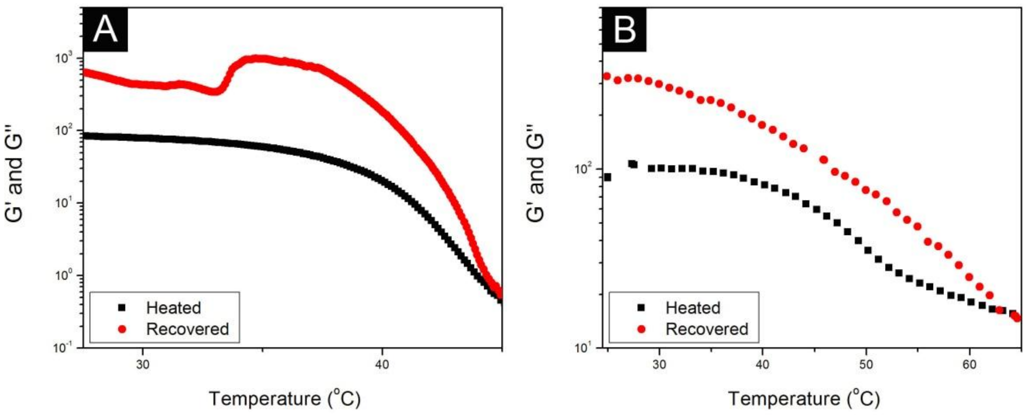

Figure S4: Temperature recovery of gel strength of the gels formed by (A) 0.8 wt% of **1a** formed at pH 7.0 and; (B) 1.5 wt% of **1d** formed by treating **1c** with 5 U/ mL phosphatase at pH 7.6.

References

[1] Ma, M. L.; Kuang, Y.; Gao, Y.; Zhang, Y.; Gao, P.; Xu, B. *J. Am. Chem. Soc.* **2010,** 132, 2719–2728. doi:10.1021/ja9088764
